# Bridging Gaps in Vaccine Access and Equity: A Middle Eastern Perspective

**DOI:** 10.3390/vaccines13080806

**Published:** 2025-07-29

**Authors:** Laith N. AL-Eitan, Diana L. Almahdawi, Rabi A. Abu Khiarah, Mansour A. Alghamdi

**Affiliations:** 1 Department of Biotechnology and Genetic Engineering, Jordan University of Science and Technology, Irbid 22110, Jordan; dlalmahdawi20@sci.just.edu.jo (D.L.A.); raabukhiarah20@sci.just.edu.jo (R.A.A.K.); 2 Department of Anatomy, College of Medicine, King Khalid University, Abha 61421, Saudi Arabia; m.alghamdi@kku.edu.sa; 3 Genomics and Personalized Medicine Unit, The Center for Medical and Health Research, King Khalid University, Abha 62529, Saudi Arabia

**Keywords:** vaccine, distribution, equity, economic barriers, global health, Middle East

## Abstract

Vaccine equity and access remain critical challenges in global health, particularly in regions with complex socio-political landscapes, like the Middle East. This review examines disparities in vaccine distribution within the Middle Eastern context, analyzing the unique challenges and opportunities across the region. It provides an overview of the area’s diverse finances and its impact on healthcare accessibility. We examine vaccination rates and identify critical barriers to vaccination, which may be particular issues in developing countries, such as vaccine thermostability, logistical hurdles, financial constraints, and socio-cultural factors, or broader problems, like political instability, economic limitations, and deficiencies in healthcare infrastructure. However, we also highlight successful efforts at the regional and national levels to improve vaccine equity, along with their outcomes and impacts. Ultimately, by drawing on the experiences of previous programs and initiatives, we propose strategies to bridge the gaps in vaccine access through sustainable financing, local manufacturing, and the strengthening of health systems. This approach emphasizes the importance of regional collaboration and long-term self-sufficiency in enhancing global health security and achieving more equitable outcomes in the Middle East.

## 1. Introduction

Our global interconnectedness exposes us to a greater risk of infectious diseases and biological hazards than ever before, whether they are naturally occurring, accidentally released, or deliberately deployed. Such threats can rapidly spread across international borders, posing serious risks to global health [1]. Global health security refers to the proactive and active measures required to protect the collective health of the global population from the threats posed by infectious disease outbreaks and epidemics [2]. Vaccines are central to these preventive efforts, playing a critical role in preventing illness, reducing mortality, and mitigating the economic and social impacts of infectious diseases. However, disparities in access persist as a significant challenge, despite calls for global collaboration [3]. Ensuring equitable vaccine distribution is a moral imperative crucial for reducing health disparities worldwide, which helps strengthen economies and control diseases more effectively [4]. The issue of vaccine access and distribution has been a topic of increasing discussion within international health and scientific circles, particularly during the COVID-19 pandemic. Some studies indicate that the success of vaccination programs varies significantly across countries, depending on their socioeconomic status. High-income countries typically have more widespread and up-to-date vaccination programs, achieving higher vaccination coverage that is rarely seen in middle- and low-income countries [3,5]. These differences include economic status, inadequate cold chain and transportation infrastructure, limited funding, insufficient public health resources, and lower vaccine acceptance [6]. Therefore, understanding and addressing these issues is essential to achieving global health equality and preventing future health crises [7]. This is particularly true in regions with complex socio-political environments, such as the Middle East, where countries within it differ considerably in socioeconomic status, political stability, and global economic importance, reflecting the region’s broad diversity [8,9]. Some nations endure starvation and war, while others experience economic prosperity, resulting in variations that profoundly impact healthcare and other sectors [10].

Previous reviews on vaccine access and equity have primarily focused on global perspectives or economic classifications, rather than regional contexts, which makes our study unique for the region. One of these articles is a study conducted by Piot et al. (2019) which emphasized an unfinished immunization agenda globally [11], while Duan et al. (2021) highlighted the relation between country income level and COVID-19 vaccination coverage [6], and Nunes et al. (2024) examined the role of global health partnerships without regional specificity [5]. Similarly, Sehovic and Govender (2021) studied the vulnerabilities in global health security during the COVID-19 pandemic but did not address the Middle East’s unique problems [7]. A few local studies included a systematic review of immunization coverage among migrants and refugees in the MENA region and found that migrants were half as likely to be vaccinated as non-migrants [12], similar to a different review by Iqbal et al. (2023), who studied COVID-19 vaccine hesitancy among parents, finding an average 44.2% vaccination rate due to safety concerns and misinformation [13]. However, these are population or theme specific and do not provide a comprehensive region-wide analysis; therefore, our paper offers that gap perspective in the Middle Eastern countries within the MENA region as classified by UNICEF, with a particular focus on Bahrain, Egypt, Iran, Iraq, Jordan, Kuwait, Lebanon, Oman, Qatar, Palestine, Saudi Arabia (SA), Syria, United Arab Emirates (UAE), and Yemen [14]. We also synthesize recent evidence on vaccine access disparities, structural barriers, and political complexities, while outlining practical, region-tailored strategies, such as local manufacturing, innovative cold chain solutions, and addressing inequalities in vaccination rates. Additional analysis of different vaccine coverage will also be conducted using quantifiable indicators, such as immunization rates and country income levels, and comparing both national and regional strategies to provide targeted suggestions that aim to improve vaccine access, uptake, and program effectiveness in the region. This article presents a data-driven perspective on vaccine distribution and equity, translating global goals into context-specific recommendations for a region deeply affected by conflict and economic inequalities, thereby bridging a significant gap in vaccination efforts.

## 2. Global Overview of Vaccine Equity and Access

**Figure 1:**
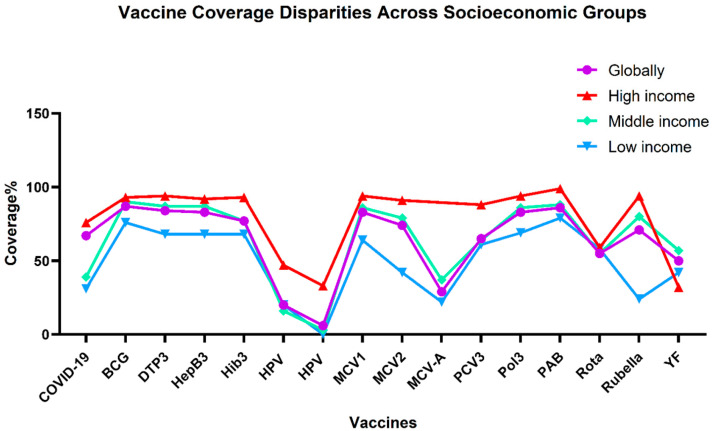
Vaccination coverage rates for different vaccines created using GraphPad Prism software version 8.0.2. It includes global rates and rates in high-income, middle-income, and low-income countries from the WHO/UNICEF Estimates of National Immunization Coverage (WUENIC) in 2023 [20,21].

Ensuring equitable vaccine distribution globally means that all nations receive fair access to vaccines, regardless of their economic status. However, vaccination rates vary between and within countries; wealthier nations access vaccines early due to their significant investments in their development and procurement, while those with lesser incomes lag with more restrictive vaccine policies and lower immunization rates [6]. While some countries face unique challenges that hinder vaccine acceptance, such as social misinformation, conflict, or political instability, issues with vaccine distribution are not exclusive to developing nations. Barriers, such as supply shortages, vaccine hesitancy, insufficient production capacity, and delivery difficulties, are faced across all income levels [11]. These shared issues underscore the complexity of global immunization efforts, which we will discuss further in the following sections. In Figure 1, vaccination rates are presented for various vaccines categorized by a country’s income level, highlighting how the coverage fluctuates across countries from different economic backgrounds. High-income countries consistently demonstrate the highest vaccination coverage, with rates that regularly exceed 90% for most vaccines, underscoring their robust healthcare infrastructure and widespread access to vaccines. The situation differs for middle- and low-income countries: low-income countries typically have lower vaccination rates for most vaccines; in contrast, middle-income countries have achieved relatively high coverage levels for certain vaccines (e.g., COVID-19, DTP3, MCV2, and PCV3). The differences are in COVID-19, HEPB3, Pol3, and Rubella. The global average is positioned midway between middle-income and low-income countries; meanwhile, the coverage in middle-income countries approaches global levels but is much lower than that of high-income nations. Low-income countries fall substantially below both, which suggests potential challenges in vaccine distribution, access, and possibly public health infrastructure challenges in middle- and low-income nations. The vaccination rates for ROTA and YF vaccines in low-income countries are interestingly higher by 2–7% than those in high-income countries, which could be attributed to various factors, such as higher disease prevalence in these regions, which leads to increased prioritization of vaccination efforts through outbreak response campaigns, or other contributing factors, like higher mortality rates, aggressive vaccination campaigns, and prioritization by international health organizations, like the support availability from Gavi during outbreaks [15,16].

In some cases, the increased vaccination rates in low-income countries are not necessarily a positive sign. Immunization coverage of infants has reached high levels in many of the poorest developing countries, where complementary strategies for HPV control, such as adult screening and treatment, are poorly developed [17]. By addressing socioeconomic barriers and implementing targeted interventions, vaccine access in low-income regions can be improved, thereby bridging the gap and achieving more equitable global health outcomes. Monitoring these disparities is crucial for tailoring programs and policies, enabling the allocation of resources to underserved individuals and communities [18]. As observed with rotavirus vaccines, the effectiveness rate can vary significantly depending on a country’s income level, which is particularly important since additional vaccine benefits would increase the overall impact on the population and improve cost-effectiveness [19]. In the following subsections, we outline the key challenges that continue to hinder vaccination efforts in developing countries, highlight emerging and evidence-based innovations designed to overcome these obstacles, and provide an overview of the critical role that international organizations and donor agencies play in expanding and sustaining immunization coverage.

### 2.1. Vaccination Barriers in Developing Countries

Developing countries face significant challenges in vaccine distribution and access, which hinder their efforts to immunize their populations. One major issue is vaccine thermostability, which requires that they be consistently stored within a temperature range of 2–8 °C to maintain their efficacy. They can become unstable when exposed to light, heat, radiation, or any other unfavorable environment [22]. In this context, the need for specialized healthcare personnel, such as pharmacists, becomes critical. They ensure proper handling, storage, and monitoring of vaccines throughout the distribution chain using their specialized knowledge in chemistry, biology, pharmacology, and pharmaceutical technology [23,24]; consequently, without their supervision or any similar ones, vaccines’ stability risks increase, particularly in regions with infrastructure gaps or with natural calamities [23]. The shortage of pharmacists is more prevalent in many low- and middle-income countries compared to high-income nations [25], making them more susceptible to cold chain failures, vaccine potency loss, and wastage due to inadequate monitoring protocols, insufficient guidelines, and a lack of trained personnel [26]. In developing countries, this issue is further complicated by their inadequate storage infrastructure (e.g., poor power supply, unreliable cold chain equipment, irregular maintenance, refrigerant leaks, and insufficient repair parts), which threatens the safety and potency of vaccines; that is, once lost, it is irreversible [22,27]. A combination of logistical and financial hurdles makes it more challenging for developing countries. Logistical challenges in vaccine delivery to target populations can be hindered by poor road conditions, adverse weather, potential theft, and the need to reach remote areas, leading to unpredictable delivery times that may compromise the integrity of the products [28]. Additionally, various factors can hinder vaccine accessibility, including unfair pricing, particularly for countries that are neither wealthy enough to afford high-cost vaccines nor poor enough to receive funding from programs, like Gavi, countries with rugged terrains, such as Nepal, Pakistan, and Afghanistan, and conflict-affected areas, like Yemen, Syria, and South Sudan, struggle with vaccine accessibility due to isolation and instability [11,29].

Additionally, socioeconomic factors influence vaccination rates across different populations. Poverty, for example, is a significant factor that often prevents people in low-income areas from accessing healthcare and affording the costs associated with vaccines, such as transportation to clinics [6]. Expectedly, education levels also influence vaccine acceptance, where lower education and income are linked to higher concerns about vaccines, as individuals often struggle to process, understand, or evaluate the risk–benefit claims of vaccines [30]. Beyond general education, health professionals, such as pharmacists, physicians, and nurses, play a crucial role in promoting health literacy and vaccine uptake [31,32]. A meta-analysis study has shown that pharmacists acting as immunizers or advocates increase vaccination rates significantly (relative risk, 1.14–1.31) compared to standard care without pharmacist involvement [31]. Likewise, nurses and physicians strongly perceive it as their duty to promote vaccination, with over 78% and 85% reporting that they educate patients in person about vaccines [32]. This highlights the fact that more informed patients adhere more consistently and confidently to vaccination, underscoring the importance of investing in both general education and health professional-led communication [33].

**Figure 2:**
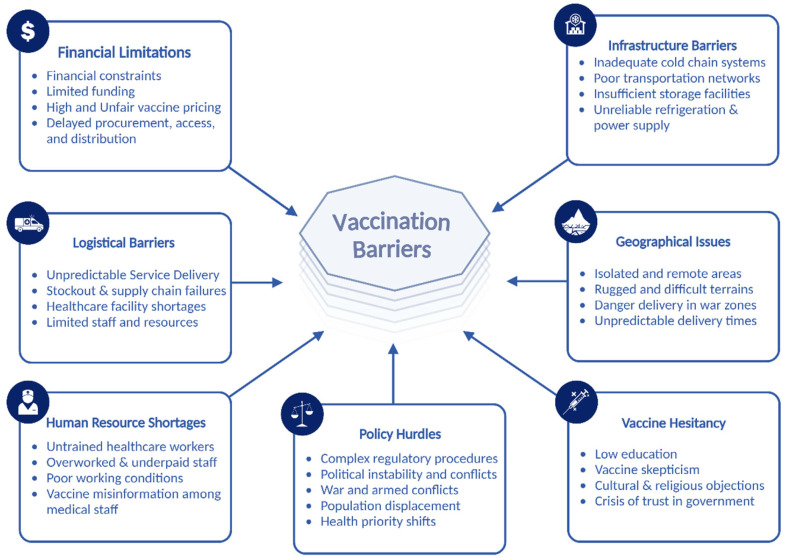
The main challenges that affect vaccination efforts in the Middle East, categorizing the barriers into financial limitations, infrastructure barriers, logistical barriers, human resources shortages, policy hurdles, and vaccine hesitancy and highlighting the multifaceted nature of the problem and the need for comprehensive solutions to improve global vaccine access and equity.

Furthermore, another crisis of trust in vaccines comes from cultural and religious perspectives; people often evaluate vaccine risks through cultural identity and group connections. However, religious objections to vaccines, like smallpox, were based on beliefs about purity and divine intervention [30]. Finally, war, violence, and armed conflicts collectively worsen all previous issues for affected populations; these crises destroy healthcare facilities, ruin health systems, cause loss of healthcare workers, disrupt vaccine cold chains, and cause people displacement; in these areas, humanitarian access is often blocked or hindered, and a shift in priorities occurs from prevention to treating casualties, leaving communities vulnerable to diseases [34]. Figure 2 provides an overview of the key barriers discussed, clearly visualizing the main challenges to vaccination.

### 2.2. Innovative Solutions for Vaccine Distribution in Developing Countries

Various strategies can improve vaccine distribution challenges and enhance immunization programs in developing countries. Innovative technologies, such as microneedle patches and heat-stable formulations, combined with the modernization of cold chain infrastructure using solar-powered refrigerators, can significantly enhance vaccine storage, transportation, and administration in resource-limited settings [35]. For example, a study by Adhikari et al. estimated that measles vaccinations using microneedle patches cost around USD 0.95 per dose, compared to USD 1.65 for traditional subcutaneous injections, and a reduced cost per case averted from USD 2.64 to USD 1.66 at 95% coverage [36]; unlike traditional injections, they are thermostable and can be stored at room temperature, which eliminates the need for cold chain infrastructure. Another case demonstrating this is the dissolving microneedle patch for inactivated polio vaccine, as they have been shown to retain over 70% antigen activity after 2 months and more than 50% after 1 year when stored at 5 °C or 25 °C [37]. Similarly, solar-powered cold chain systems have been introduced in resource-limited settings to maintain vaccine potency during power outages; since 2017, Gavi has installed over 66,000 solar-powered refrigerators worldwide, with approximately 60% operating on direct-drive solar systems to support immunization efforts [38]. Moreover, according to a WHO/UNICEF evidence brief, solar direct-drive systems are now widely recommended for off-grid use, reliable cold chain maintenance (+2–8°C), reduced maintenance by removing batteries, and resilience in remote environments [39]. While their capital cost (USD 650) is relatively high, their exceptionally low operating cost (USD 32) makes them more economical over time in remote or conflict-affected areas compared to fuel-based alternatives, like LPG or kerosene, which have much higher running expenses [39].

Additionally, mobile vaccination units and drone technology can help overcome road transportation disruptions, reach remote areas, and facilitate emergency last-mile distribution [28]. However, strict cold chain protocols must be maintained to ensure vaccine stability during drone transport, using insulated payloads equipped with real-time temperature and humidity monitors. This ensures that vaccines remain within the 3–4 °C range, despite fluctuations in conditions, such as humidity, wind, or heat, during flight [40]. Regarding legal frameworks, drones have been successfully utilized for medical logistics in countries, such as Rwanda and Ghana, operating under structured guidelines. However, many Middle Eastern countries still face regulatory gaps concerning airspace use, data privacy, and liability in the event of cold chain failure. For example, a recent study from Jed-dah found that, although drones were allowed, users’ primary concerns with their delivery included privacy and security risks [41]. In contrast, the Oman Civil Aviation Authority (CAA) and Egypt’s Ministry of Defense, both allow drones but with stringent regulations that emphasize safety and insurance with violations punishments but include no specific guidelines on medical payloads or health-related use cases [42,43]; which highlights their hesitancy in using them, with no official unified framework yet governing medical or vaccine drone delivery in the region.

At the national level, efficient management and planning of procurement, maintenance, and replacements, along with stakeholder collaboration to enhance regulatory systems and communication, can help improve immunization program efficiency in low- and middle-income countries [27]. Innovative approaches to vaccine programs and coordinated global funding efforts, rather than relying solely on market-driven interest, could benefit middle-tier economies that cannot obtain funds or afford vaccines [11]. Community engagement strategies can improve vaccine acceptance among populations, including the use of early warning systems, coordinated responses, real-time information sharing, innovative approaches, behavioral demonstrations by cultural or public health leaders, and school- and community-based programs with mobile clinics [6,44,45]. Nevertheless, the effectiveness of national immunization programs shows wide variation in the Middle East, which is closely tied to the strength of National Medicines Regulatory Authorities (NMRAs), being the primary responsible for ensuring the quality, safety, and efficacy of medicines and other health products [46]. Countries, like SA and Egypt, have reached WHO maturity levels 3 and 4, respectively, demonstrating robust regulatory oversight; the Saudi Food and Drug Authority (SFDA) is considered one of the most advanced regulators in the region, and Egypt’s Drug Authority (EDA) has been recognized as the first in Africa and the Middle East to reach maturity level 3 in medicine regulation [47,48]. In contrast, several other countries, including Yemen and Palestine, are still in the initial phase of implementing and developing pharmacovigilance systems, operating with fragmented or under-resourced systems that create gaps within the health system and severely affect patients [49,50,51]. Ultimately, expanding conflict-sensitive strategies for war-affected areas, particularly for the non-camp population, and implementing international agreements that protect healthcare facilities, workers, and essential services in the most troubled regions would significantly aid those in need [34].

### 2.3. International Organizations and Donor Support as Vaccination Facilitators

**Figure 3:**
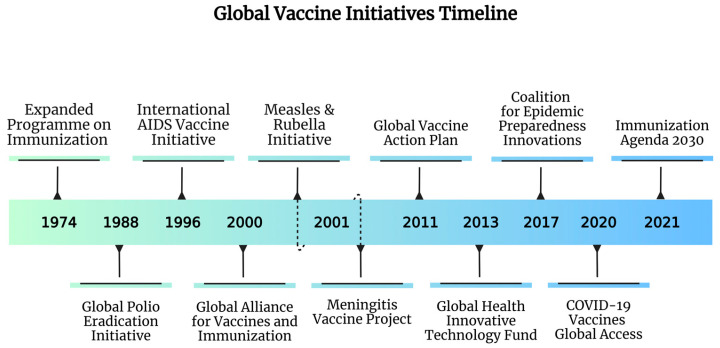
An overview of key global vaccine initiatives from 1974 to 2021, highlighting the launch and progression of critical programs aimed at enhancing immunization coverage, combating vaccine-preventable diseases, and addressing emerging global health challenges.

Vaccine-preventable diseases continue to pose a significant public health challenge, particularly in low- and middle-income countries. In recent years, remarkable progress has been made in vaccine development and program expansion, led by international organizations, initiatives, and donors working towards a healthier world [52]. These organizations play a vital role in advancing global immunization efforts by supporting activities that improve vaccine access, delivery, and coverage worldwide. For instance, the well-known World Health Organization (WHO) established the Expanded Programme on Immunization (EPI) to support routine vaccinations in developing countries [52]. The United Nations Children’s Fund (UNICEF), a primary donor for many developing countries, focuses on vaccine procurement and monitoring to achieve universal immunization, especially for children [52]. It supported the introduction of the pneumococcal conjugate vaccine in 80% of Gavi-eligible countries, which prevented millions of childhood deaths [52,53]. The Global Vaccine Action Plan (GVAP) unified vaccination goals set by the World Health Assembly (WHA) and WHO Regional Committees into ambitious targets for global immunization, which successfully advanced the introduction of new vaccines in many countries and contributed to the elimination of diseases, such as polio [54]. A final example is the Immunization Agenda 2030 (IA2030), which was launched in 2021 to guide global immunization efforts through the next decade; this aimed to reduce mortality and morbidity from vaccine-preventable diseases for everyone, emphasizing the importance of reaching “zero-dose” children—those who have never received a single vaccine dose—and integrating vaccination into primary healthcare systems by 2030 [55]. In response to the COVID-19 crisis, international health partnerships adapted rapidly to confront global immunization challenges. For instance, the Coalition for Epidemic Preparedness Innovations (CEPI) guided the acceleration of vaccine development, while Gavi, WHO, and CEPI co-led the COVAX Facility to promote equitable vaccine distribution. Based on global solidarity, COVAX includes self-financing and funded countries, aiming to support vaccine development and procurement for 20% of the world’s population [5]. However, while global initiatives are vital for vaccine distribution, encouraging self-sufficiency in developing countries is equally important [54,56]. Therefore, ensuring that local capacity is met is seen as a necessary step for international agencies to take to reduce import dependence; this involves encouraging independent vaccine procurement, developing indigenous capabilities, and investing in regional infrastructure [54]. While donations address short-term needs, long-term equity requires community-engaged vaccination programs tailored to specific contexts, such as delayed delivery and short expiration dates, especially in countries with strained health systems [52,55]. Ultimately, by prioritizing these elements, countries can enhance their capacity to respond to health crises and achieve greater equity in global vaccine distribution, thereby advancing justice and public health outcomes worldwide [57]. To better understand the initiatives and programs, refer to the summarized timeline in Figure 3.

## 3. Overview of the Middle Eastern Region

**Figure 4:**
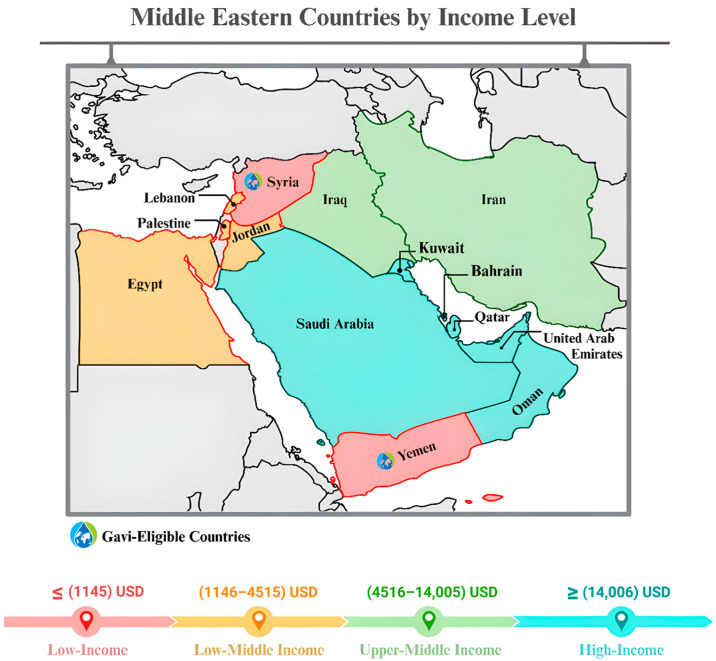
The map categorizes Middle Eastern countries by income level, using a color-coded system to illustrate regional economic disparities. Low-income countries (red) include Yemen and Syria, both of which are Gavi-eligible for vaccine funding. Lower-middle-income countries (orange) are Egypt, Jordan, and Palestine, while upper-middle-income countries (green) include Iraq, Iran, and Lebanon. Finally, high-income countries (blue) include SA, the UAE, Qatar, Bahrain, Kuwait, and Oman. Additionally, grey shading indicates non-target countries while Gavi-eligible countries are marked with a special symbol, indicating their qualification for global vaccine funding support.

Land and sea routes where Africa, Asia, and Europe meet have been historically significant due to their deeply indented coastline, connecting the regional countries to the rest of the world. This connectivity is pivotal in facilitating maritime trade, influencing control over ports and strategic seaways that alternately support and disrupt commerce over time, remaining a crucial source and destination for major economic activities and travel to this day [58]. The countries in the region share a similar geographic location and culture, yet vary in population size, health status, healthcare system, public healthcare expenditure, and epidemiological parameters [59]. The group of countries is diverse and not easily defined; it is generally grouped into high-, middle-, and low-income countries based on their economic status (Figure 4). According to WHO, this significantly affects the introduction of vaccines into national immunization programs, given its relation to many factors, including the cost-effectiveness for policy implementation [59]. Economically prosperous countries in the Middle East (e.g., Saudi Arabia, the United Arab Emirates, and Qatar) play a crucial role in providing financial aid. A clear example of their contribution is their support in giving foreign aid to Egypt [60]. From a focused standpoint, the UAE provided financial and medical supplies to Syria and multiple hospitals in Jordan. Thus, during the COVID-19 pandemic, SA funded several states in the region—especially those severely impacted by the pandemic, such as Iraq and Yemen—and Kuwait provided medical and humanitarian support to countries, like Lebanon, Iraq, and Palestine [60,61]. This demonstrates how their economic strength enables them to make significant contributions to regional stability and development.

### 3.1. Vaccine Landscape in the Middle East

**Figure 5:**
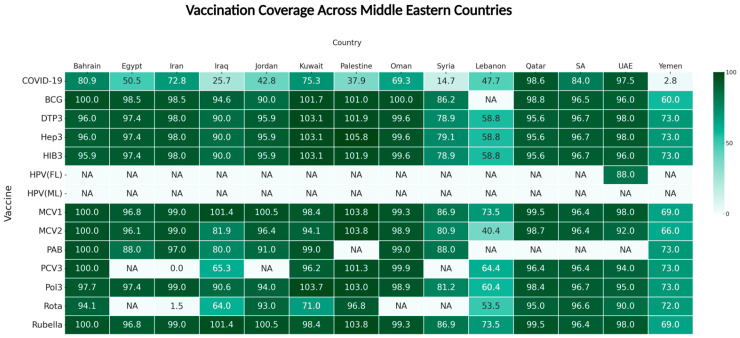
A heat map of vaccine coverage percentages across various vaccine types and countries in the Middle East, utilizing data from the WHO Immunization Data 2024 [20]. The color intensity indicates the level of coverage, with darker green representing higher coverage and lighter colors representing lower coverage.

Data from Middle Eastern countries—including Bahrain, Egypt, Iran, Iraq, Jordan, Kuwait, Lebanon, Oman, Palestine, Qatar, SA, Syria, the UAE, and Yemen—reveal distinct vaccination disparities. High-income countries have achieved near-universal immunization, whereas middle-income nations display varying levels of coverage; meanwhile, low-income countries struggle with limited resources, political instability, and weak healthcare infrastructure. These patterns in the Middle East mirror those globally, illustrating how economic and infrastructure disparities influence vaccine access. The gap between high, middle, and low-income nations highlights the need for targeted interventions to improve vaccine coverage, especially in lower-income regions. Figure 5 show the vaccination coverage across Middle Eastern countries (2024), which revealed significant disparities in vaccine uptake between high-, middle-, and low-income nations. High-income countries, like Qatar, SA, and Bahrain, consistently achieve rates beyond 95%, reflecting their developed healthcare systems and early access; conversely, middle-income countries like Egypt and Iran maintain high coverage rates for most vaccines with a notable dip only for COVID-19 vaccination, but this is not the case for all the middle-income countries. Other areas, such as Jordan, Lebanon, Palestine, and Iraq, exhibited lower vaccination coverage compared to high-income nations, highlighting the regional disparity. Finally, Low-income countries, including Yemen and Syria, faced the most significant challenges, with markedly lower coverage rates across most vaccines, particularly for COVID-19, where coverage can be as low as 2.26% in Yemen. This disparity within the region is not only shaped by income level but also by differences in governance, health system efficiency, and political status. Giving a comparative example, Oman and Bahrain differ in implementation strategies despite both being high-income countries; Oman focused on school-based vaccination campaigns and robust regional outreach to eliminate measles and rubella [62]. However, Bahrain achieved its vaccination goal by focusing on electronic health monitoring. It targeted reminders, utilizing digital infrastructure [63], highlighting how pathways to success differ based on local context and policy decisions, even among countries within the same income group. Also in the middle-income category, Egypt’s National Immunization Days, conducted with WHO and UNICEF support, have helped it maintain over 97% polio vaccination coverage, which demonstrates the power of sustained international collaboration and strong local execution [64,65]; conversely, while Jordan and Lebanon are economically comparable, reports in Lebanon significantly lower coverage for several vaccines due to different challenges they face, like political instability, heavy refugee burden, or financial crisis [66,67,68]. Looking beyond the region, Rwanda and Vietnam offer instructive models, although they are considered lower-middle-income countries [69]. Rwanda’s success stems from embedding immunization into broader maternal–child health platforms and deploying trained community health workers to track and deliver vaccines at the village level, achieving high coverage [70]. Similarly, in Vietnam, local health authorities planned and conducted outreach vaccination to maintain DTP3 rates above 90% [71]. These simple examples reinforce the idea that countries can outperform even wealthier nations if supported by well-structured immunization programs. As vaccination coverage varies significantly across regions and is not always aligned with income levels, as seen in Nepal and Palestine, this is a crucial consideration. However, both are classified as lower–middle-income countries [69], yet their immunization outcomes differ notably. In Nepal, they overcome their geographic and socioeconomic barriers through consistent commitment and community-driven efforts, preserving over 90% routine immunization coverage [72]; inversely, Palestine struggled to maintain sufficient coverage levels due to political instability and other significant vaccination challenges, especially by mid-2021, COVID-19 vaccine coverage dropped to 5% of residents in the Gaza Strip and 16% in the West Bank, with Gaza figures falling well below the 10% mark, which placed Palestinian coverage far behind the global average for its income group [73,74,75].

### 3.2. Innovative Solutions for Vaccine Distribution in the Middle East

As we discussed before in Section 2.2, where innovative solutions were highlighted as critical to overcoming vaccine distribution challenges in developing countries, a similar approach can be applied when examining the Middle East. However, the region faces unique political, economic, and infrastructural barriers, and several countries have implemented innovative strategies to address these issues. In Lebanon, over 1000 solar-powered direct-drive refrigerators were deployed across more than 800 health facilities through a collaboration between the Ministry of Public Health (MoPH) and UNICEF, significantly reducing vaccine wastage and ensuring reliable cold chain maintenance despite frequent electricity blackouts [76]. Additionally, in the UAE, the Abu Dhabi Department of Health has collaborated with the UAE’s General Civil Aviation Authority to pilot medical drone delivery across its facilities and laboratories. However, formal healthcare-specific drone regulations (e.g., for vaccines) are still being developed [77]. Additionally, the Gulf countries have contributed significantly to regional vaccine resilience; for example, the UAE’s establishment of Hayat Biotech, which was the first local COVID-19 vaccine producer in the area, along with SA’s Sadeer City project developed domestic vaccine production capabilities, both aimed at reducing dependency on imports and strengthening long-term supply security [78,79]. Bahrain’s digital health and SMS reminders, including an electronic mobile-based platform and SMS reminder services via its Ministry of Health e-services, could be used as reminders to improve vaccination uptake [80,81]. Collectively, these initiatives demonstrate how technological and strategic innovations are being leveraged across the Middle East to overcome distribution barriers; even though not all countries in the region have adopted these measures at the same scale or with equal success, it is still a step toward enhancing vaccine accessibility and building a more self-reliant and resilient immunization infrastructure.

## 4. Challenges and Disparities in Vaccine Access in the Middle East

As previously mentioned, disparities in vaccine coverage between countries have a significant impact on global public health. This section will highlight the diversity of developing and developed countries, primarily addressing the political, economic, and healthcare challenges and their impact on vaccine accessibility in the Middle East.

### 4.1. Structural and Political Challenges

Being the most conflict-affected region in the world, the Middle East is today marked as one of the most unequal areas, characterized by high levels of political instability, including wars, invasions, and revolutions [82,83]. Regional conflicts also involve neighboring countries; for example, SA tentatively supported Iran until 2016, but the relationship between them was disturbed in 2016 due to different regional interests in Yemen, Iraq, Lebanon, and Syria [84]. A more recent event occurred during the COVID-19 pandemic, when some countries were still recovering from war with fragile infrastructure; Iraq experienced hospital fires in 2021, and Yemen was going through the world’s worst humanitarian crisis [84]. Another unfair situation arises from the Palestine–Israel political tension, where Israel controls Palestine’s borders and land. This control has led to vaccination issues, like the arrival of expired COVID-19 vaccines to the Palestinian population due to the ongoing conflict, which negatively impacted public health [85]. Poor immunization coverage in conflict zones leads to outbreaks of vaccine-preventable diseases (VPDs); for instance, Yemen is severely affected by the ongoing civil war and has the lowest regional coverage, a situation further exacerbated by inadequate immunization services, even in urban areas [86]. In Syria, the healthcare system has been severely disrupted by facility destruction, lack of medical staff, and critical shortages of essential medications, leading to the re-emergence of diseases, like tuberculosis, measles, and polio [87]. Another lies in the vaccine’s cost-effectiveness, as its price continues to increase. However, resources remain limited in some countries in the region, posing a challenge to the vaccine procurement process and affecting coverage [58]. Under normal circumstances, high-income countries, like the GCC (Gulf Cooperation Council) nations, do not typically face significant constraints in financing their immunization programs; however, the decline in oil prices has exposed their vulnerability and heavy dependence on oil and gas production [88,89]. Gulf countries face several health-related challenges, including high medical expenses and difficulties in meeting the healthcare needs of their non-national migrant populations. Despite the urgent healthcare needs of migrants, Gulf states prioritize their citizens, who receive healthcare benefits that migrants are excluded from [90]. Middle-income countries ineligible for Gavi support lag in introducing new vaccines despite their critical need, as they report paying relatively higher prices than similarly situated countries. Countries eligible for Gavi funds, such as Yemen and Syria, face substantial governmental challenges and instability that hinder their progress toward vaccination objectives [58]. In addition to domestic political and economic issues, international diplomacy through Global Health Diplomacy (GHD), and global institutional relationships particularly impact vaccine equity [91,92,93], with countries that have stronger diplomatic ties to multilateral organizations, such as the World Bank and WHO, are often better positioned to access vaccine funding and other logistical support [4]; however, politically isolated or conflict-engaged nations, like Syria, Yemen, or parts of Palestine, are often deprioritized in global vaccine campaigns or face restrictions in accessing aid mechanisms, like COVAX [94,95]. These imbalances in international engagement further compound vaccine inequity in the region.

### 4.2. Regional and Social Disparities in Access

The Middle East exhibits the lowest levels of public spending on healthcare, which can be related to the uneven distribution of healthcare resources [96]. Low-income countries in the region struggle with deteriorating healthcare infrastructure, shortages of essential medications and supplies, and inadequate coverage in remote areas [96]. These problems are accentuated in countries with ongoing conflicts (e.g., Gaza, Iraq, Yemen), exacerbating health outcomes, particularly in maternal and child health; for instance, Palestine achieved only 10% COVID-19 vaccination coverage in June 2021, 50% less than Israel, revealing significant challenges in healthcare capacity and vaccine availability compared to neighboring countries [85,96]. An essential factor is religious beliefs, which affect vaccine refusal, especially among conservative Muslim communities, where different concerns exist related to ethical dilemmas or interfering with natural processes associated with reduced immunization rates [61]; for example, outbreaks of preventable diseases, like polio, diphtheria, and measles, occur in Muslim-majority countries. Furthermore, low education affects the healthcare system, as in SA, studies assessing COVID-19 vaccine hesitancy revealed that individuals with lower incomes and educational levels reported higher opposition to vaccination [61]. Together with international reporting, local health actors have provided valuable insights into the barriers they face on the ground. In Palestine, the Ministry of Health and Médecins Sans Frontières in Gaza cited logistical delays, cold chain failures, and distrust in public health messaging as key contributors to low vaccine uptake in the field assessment reports [97,98]. In rural Yemen, frontline workers faced armed conflict zones and infrastructure collapse, as they reported how delivery routes were blocked and unreliable [99]. Similarly, in Jordan, national campaigns struggled to reach marginalized communities, including refugees living outside formal camps, despite coordination between the WHO and MOH [100]. Similar to Iran, they faced shortages of human resources, inadequate financing, and a lack of medical supplies and equipment, particularly in remote and underserved areas, which significantly impacted the COVID-19 vaccination rollout [101]. Also in Lebanon, community engagement reports and monitoring by local organizations have noted vaccine hesitancy linked to low public trust in authorities, along with severe disruptions in cold chain storage due to the country’s power crisis [102]. These experiences highlight how global vaccine data may mask subnational disparities and local implementation challenges.

### 4.3. Opportunities and Initiatives to Improve Vaccine Equity

The Middle East has seen various innovative initiatives focused on enhancing vaccine equity and access to improve public health at national, regional, and global levels, addressing unique community challenges. This section will examine some of these initiatives, highlighting key programs, their implementation strategies, and their notable achievements. Table 1 provides an overview of significant regional initiatives, outlining their objectives and outcomes.

**International support and collaboration.** Vaccine equity in the Middle East indeed was improved through international partnership collaborations (like WHO and UNICEF) with national governments; for example, the Egyptian polio program, which combined efforts of WHO, UNICEF, CDC, and USAID, has succeeded in maintaining 97% vaccination coverage and a polio-free status since 2006. In vulnerable areas, such as Syria and Yemen, Gavi provided funds to introduce new vaccines and strengthen the healthcare system [9,10,65]. The Global Vaccine Action Plan (GVAP) was implemented from 2016 to 2020 through the Eastern Mediterranean Vaccine Action Plan (EMPVAP), prioritizing immunization programs, equitable access, and sustainable funding, which led to the elimination of rubella and measles from Oman and Bahrain [113,114]. In Jordan, however, a remarkable effort was seen from UNICEF and various national governments in combating vaccine hesitancy. An initiative called “Shabab Elak o Feed” aimed to help the elderly register for COVID-19 vaccinations and correct misinformation [115]. Bilateral agreements during the COVID-19 pandemic have furthered vaccine development in the region; SA and the University of Oxford collaborated on the ChAdOx1 vaccine against MERS-CoV, which has moved to human trials [107]. The Hayat Biotech company, a joint venture between the UAE and China, produced the first Middle Eastern COVID-19 vaccine, Hayat-Vax, which was distributed in 64 countries and has a production capacity of 200 million vials with approximately 80% vaccine efficacy [79,110,111]. Gulf countries have also made significant contributions to international vaccination efforts through funding and donations, supporting the COVAX initiative with USD 221 million between 2020 and 2022 [116]. In April 2024, SA committed USD 500 million to the Global Polio Eradication Initiative, aiming to vaccinate 370 million children annually over the next five years [117].

**Regional and National Initiatives.** The Middle East faced significant challenges during the COVID-19 pandemic, underscoring the need for regional cooperation to achieve vaccine equity among countries. The Group Purchase initiative (1978) was a particularly effective collaboration between Gulf countries, resulting in numerous advantages, including cost savings, ensuring an adequate and continuous supply of vaccines, and accelerating the introduction of new vaccines into immunization programs [103]. However, the Arab League also demonstrated some collaborative efforts as they signed a Memorandum of Understanding (MoU) with Gavi, promising to improve immunization coverage through education and societal involvement, as well as enhancing women’s and children’s health by scaling up immunization programs in member countries between 2016 and 2020 [118]. Furthermore, the national vaccine initiatives are essential for addressing country-specific challenges and improving vaccination rates, particularly in countries with large populations. For example, Lebanon and Iraq faced serious issues with their refugee populations; in Lebanon in 2021, they adopted an inclusive approach to give the COVID-19 vaccine to millions of displaced Syrian and Palestinian populations using a vaccination campaign. It ensured that refugees received equitable access to vaccines, supported by international organizations, the World Bank, and NGOs [109]. In 2015, the Ministry of Health in Iraq implemented a cholera vaccine initiative targeting approximately 255,000 refugees with two doses of oral cholera vaccine during a widespread outbreak, achieving 87% coverage and demonstrating the feasibility of such campaigns in conflict settings [119]. In addition, the UAE was the first Middle Eastern country to include the HPV vaccine program in its national immunization program, with a vision to reduce cervical cancer rates significantly over time despite the cultural and religious challenges this vaccine bears. Utilizing the tech decade to their advantage, they launched the #TogetherWeRecover campaign in 2021 to address COVID-19 vaccine hesitancy through social media and public messaging, resulting in high vaccination rates [105,111]. A critical remake towards vaccine security and long-term self-sufficiency in the Middle East is the establishment of regional vaccine manufacturing facilities, such as the Sadeer City vaccine and vital medicines factory in SA; this trend extends beyond SA, with Iran and Egypt also developing capabilities for local production of traditional vaccines [10,112]. All previous examples showcase successful initiatives and programs in the Middle East that point out the potential for broader regional cooperation and future initiatives to further enhance equitable vaccination in the region and hopefully for countries in similar situations. Additional details of income level, peace index ranking, major funding sources, and Gavi eligibility for Middle Eastern countries are summarized in Table 2. 

**Mechanisms of Religious Law’s Impact on Vaccine Policies**. Several Muslim-majority countries have faced challenges related to the intersection of religious jurisprudence and vaccine acceptance and policy, often stemming from perceived permissibility under Islamic law or cultural interpretations of morality [138]. A parallel case between SA and Iran provides further context, where vaccines, like meningococcal, yellow fever, or poliomyelitis, were mandatory in SA for pilgrims and was widely accepted as they aligned vaccination policy with Islamic principles of communal harm prevention, which substantially reduced disease outbreaks during these religious gatherings and prevented the spread among returning travelers [139,140]. In contrast, Iran faced resistance to HPV vaccination, disclaiming it as an encouragement to premarital sexual activity, which is a concept contrary to cultural and religious norms [141]. The acceptance of this vaccine improved after Fatwas and religious endorsements clarified that it was permissible and necessary for cancer prevention across several Muslim countries facing similar issues [138]. The same situation occurred in Egypt, where Hepatitis B vaccine uptake was low due to stigma linking the disease to sexual activity and drug use. The country subsequently reframed the vaccine as essential for liver health and infant protection, supported by statements from the WHO emphasizing disease prevention as a moral duty [142,143]. Collectively, these cases underline the importance of integrating religious values and cultural sensitivity into immunization strategies to overcome hesitancy and promote vaccines.

### 4.4. Actionable Strategies and Policy Recommendations

Improving vaccine equity and access in the Middle East is a challenging yet achievable goal that requires a comprehensive approach. The gap in vaccine availability across diverse economic landscapes is considered one of the region’s main issues, making it essential to develop sustainable financing mechanisms for consistent vaccine access. A key strategy for this is investing in local vaccine manufacturing capabilities, reducing dependency on imports, lowering costs, and enhancing regional self-sufficiency, as seen in the UAE with their Hayat Biotech or SA vaccine manufacturing facility. This approach offers long-term benefits in terms of supply stability and economic growth but may require considerable initial investment. Considering that vaccination coverage is influenced by more than just a country’s economic status, it also depends on the specific vaccination strategies adopted. For example, countries may adopt various vaccination strategies depending on their needs, such as the continuous strategy case [144]. Factors such as policies, delivery challenges, acceptance rates, and cultural and educational elements also significantly impact vaccination rates, regardless of the economic level [145,146]. Generally, high-income countries have an opportunity to play a critical role in regional development, particularly GCC nations, where they can invest in vaccine research, development, and production facilities in lower-income neighboring countries, like Jordan or Yemen, which not only aids in regional development but also strengthens diplomatic ties and enhances overall health security; global initiatives, such as the African Vaccine Manufacturing Initiative [147], place the Gulf as a leader in regional health security and strengthening local structural frameworks, while aligning with WHO recommendations for high-income nations, which suggest the allocation of at least 0.1% of their GNI (or GDP) to health aid, making it a well-established benchmark for official development assistance supporting immunization efforts in low- and middle-income countries [148]. They should also consider establishing dedicated funds for vaccine equity, focusing on sustainable solutions rather than short-term aid; it could include training programs for healthcare workers, support for cold chain infrastructure development, and technology transfer initiatives for low-income countries and conflict-affected areas, besides the possible combination of international financial support and the capacity-building initiatives they can have. Additionally, middle-income countries face particular challenges as they often do not qualify for international aid, such as Gavi support. Creating tiered pricing systems can help these countries access affordable vaccines as needed. Their public–private partnerships can also boost investment in the vaccine sector, promoting innovation and reducing costs. Equally important is strengthening health systems across the region, which involves developing reliable financing structures that ensure affordable, high-quality healthcare for everyone (e.g., citizens, residents, refugees, and displaced individuals). Also, comprehensive policies with a solid financial foundation can address additional barriers, such as providing shuttle services or travel refunds to boost vaccine uptake in remote areas. Though it is not just about money, investing in people and infrastructure by developing healthcare workers’ skills and improving healthcare facilities can support the long-term success and sustainability of the region’s health systems. Education and awareness also have an essential role and a significant impact; implementing regular training programs for healthcare professionals and students can build a strong foundation of knowledge about vaccine safety and efficacy. This educational effort aligns with the need for stronger regional cooperation, particularly in conflict zones where protecting healthcare facilities and personnel is a top priority. Community engagement stands out in this area with local leaders addressing specific community needs, embracing culturally sensitive approaches, and establishing partnerships with community organizations. This can help build more comprehensive trust and health awareness that reaches vulnerable populations, leveraging their established connections. In addition, by integrating early warning systems, such as those in Bahrain, and addressing vaccine concerns through familiar channels, we can create a more effective and culturally appropriate vaccination strategy. This approach can foster a more comprehensive understanding of trust and health knowledge that may influence nearby countries with similar cultures or religions. Looking forward, several specific strategies and structural advantages that large global players, such as the U.S. and China, implement in vaccine development, manufacturing, and distribution are currently lacking in most Middle Eastern countries. They have dedicated vaccine research institutes, such as the U.S. National Institutes of Health (NIH) and China’s National Vaccine and Serum Institute [149,150], along with major investments in biotechnology and strong partnerships between universities, government, and private industry, such as the U.S. collaboration with Moderna during COVID-19 vaccine development [151,152]. Another real-life example is the Strategic National Stockpile, which enables the country to maintain buffer supplies of vaccines and critical raw materials [153]. This approach could greatly benefit the Middle East, particularly for high-demand technologies, such as mRNA platforms. Similarly, the FDA (U.S.) and NMPA (China) have developed fast-track approval pathways for emergency vaccines, enabling the accelerated deployment of these vaccines during public health emergencies [154,155]. In contrast, regulatory agencies in the Middle East often lack harmonized processes and expedited approval mechanisms, resulting in delays and a limited regional capacity for rapid vaccine rollout. The region needs an aggressive pursuit of licensing agreements for mRNA, viral vector-based, and other novel platform technologies, along with comprehensive capacity-building initiatives for biomanufacturing and regulatory expertise; closing these gaps requires substantial investment in local research, advanced manufacturing facilities, regulatory harmonization across Middle Eastern countries, and long-term partnerships for technology transfer and raw material security. Despite being challenging to achieve, these goals would represent a paradigm shift for the region, one that safeguards public health, drives scientific and economic development to protect millions of lives through timely and self-sufficient vaccine solutions.

## 5. Bridging the Gaps for Improving Vaccine Access

**Figure 6:**
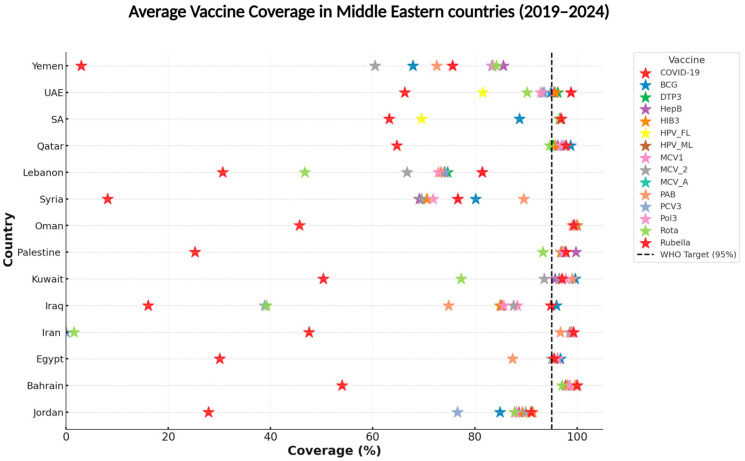
The chart illustrates the mean vaccination coverage across multiple vaccines for Middle Eastern countries from 2019 to 2024 [20]. Each star represents the average coverage of a specific vaccine in a country, and the dashed vertical line at 95% indicates the WHO-recommended target for vaccine coverage. While several vaccines approach or exceed this threshold, notable gaps exist between them.

Although the Middle East has learned several important lessons from recent experiences and studies, it still faces complex challenges in achieving vaccine equity. It requires cooperative support to address and resolve them. The COVID-19 pandemic underscored the importance of cross-border cooperation and the necessity for sustainable regional coordination programs, particularly in conflict-prone areas, such as the Palestinian territories. Lessons from the pandemic suggest that policymakers must respect and protect the fundamental human right to health regardless of nationality or background. At the same time, Global Health Initiatives should shift their focus from commodities to health systems support in fragile states, address the acute shortage of professional human resources, and strengthen healthcare infrastructure [85,86]. Improving vaccine equity also requires addressing the political aspects of the vaccine innovation system, including technology transfer and manufacturing capacity in low- and middle-income countries; middle-income countries in the region that are ineligible for Gavi support struggle to introduce new vaccines due to limited resources. These countries lag WHO recommendations for introducing necessary vaccines, like HPV, PCV, and Rotavirus vaccines [58,96]. This benchmark is designed to ensure herd immunity, which protects entire populations from highly contagious diseases, such as measles and polio, or future pathogens [156]; therefore, when the coverage drops below this threshold, communities may face heightened risks of outbreaks that can spread rapidly across borders especially in a region characterized by high population mobility and humanitarian crises, like the Middle East. In Figure 6, immunization data from the WHO (2019–2024) were collected; see Supplementary Materials for a detailed country-level vaccine coverage.

Furthermore, the average vaccine coverage was calculated for multiple essential vaccines across Middle Eastern countries to compare it with the WHO target of 95% [156]. The graph highlights significant disparities within the region, where Gulf countries like the UAE and Qatar are nearly on track. In contrast, many others, such as Yemen, Syria, and Iraq, continue to fall far below, particularly in terms of COVID-19 and HPV vaccine coverage. These gaps stem from conflict, fragile health systems, and disruptions in supply chains, compounded by vaccine hesitancy and inequitable access for vulnerable groups; therefore, minimizing these issues would significantly reduce the risk of large-scale outbreaks, strengthen regional health security, and contribute to achieving the WHO Immunization Agenda 2030 goals; this is possible by implementing actionable strategies like the ones mentioned before in this article, which would accelerate progress toward universal vaccine coverage and build resilience against future health crises.

## 6. Study Limitation

Although the WHO is a trustworthy and authoritative source for vaccine-related data, gathering accurate and reliable data is generally challenging due to regional conflicts, the refugee crisis, and other political turmoil in the region [157,158]. This is especially evident in the more remote areas of various conflict zones, such as Yemen and parts of Syria [159,160]. This also extends to the undocumented refugees who are mostly omitted from any government vaccination initiative [161]. Additionally, a correlation has been demonstrated between low public trust and falsely elevated vaccine coverage, suggesting that some countries may have inflated data that fail to accurately represent the actual real-world vaccination rates, further undermining the reliability of specific data [162]. Another limitation lies in the inability to verify national vaccination coverage data independently, as many Middle Eastern countries do not publicly share disaggregated or raw immunization datasets on their government websites, leaving researchers to rely solely on WHO vaccination rates, with no secondary validation source.

## 7. Conclusions

The Middle East has demonstrated an encouraging ability to collaborate on past vaccine initiatives, proving its potential to overcome regional difficulties for public health. However, current efforts to improve vaccine access and equity fall short. While international aid plays a vital role, a long-term vision for regional self-reliance is crucial. Before becoming a global vaccine hub, the Middle East must navigate its internal complexities and develop the capacity to tackle future infectious diseases. Ultimately, ensuring the health and safety of Middle Eastern countries is a shared responsibility, which requires a collective effort involving solid regional collaboration and commitment from policymakers, public health professionals, and the public, alongside continued support from the global health community. The Middle East can transition from a vaccine consumer to a producer, creating a more equitable and self-reliant distribution system. This shift addresses immediate health needs and contributes to long-term economic stability and regional cooperation. Combining these solutions can enhance vaccine equity and access in the Middle East across various economies, particularly for those who need them most. Of course, the aim is not only to increase vaccination rates but also to enhance global health security, leading to a better life for everyone and better preparedness to face future health crises, thereby contributing to global well-being.

## Figures and Tables

**Table 1:** Vaccination initiatives and programs across Middle Eastern countries.

Initiative	Timeline	Countries Involved	Summary	Key Outcomes	Supporting Data/Statistics	Reference
Vaccine Group Purchasing Program	1985—present	Bahrain, Kuwait, Oman, Qatar, SA, the United Arab Emirates, and Yemen (since 2004)	In 1978, the Gulf states initiated a joint drug tender, which evolved into a group purchasing system for medical products. The group purchasing of vaccines started in 1985 and required member countries to purchase at least 60% of their vaccine needs through the program.	Achieved savings in vaccine and personnel costs. Ensured an adequate and continuous supply of vaccines. Accelerated the introduction of new vaccines.	In April 2003 cost savings ranged from 4 to 46% per vaccine compared to local tenders. SA procures 100% of its public sector vaccines via the program; others ~80%. Immunization coverage (DPT3) across GCC countries reached 93–98% in 2001.	[103]
Meningococcal Vaccination for Hajj and Umrah Pilgrims	1987—present	SA	Following the 1987 MenA outbreaks, mandatory MenA-C vaccination was implemented for Hajj pilgrims and visitors. Despite this, invasive meningococcal disease (IMD) outbreaks persisted, with notable cases occurring between 1995 and 1999 and 2000 and 2001, mainly due to MenW. From 2002, MenACWY vaccination became mandatory, later switching to a conjugated vaccine. Immunization efforts also expanded to include Saudi residents and infants.	Significant decrease in IMD outbreaks. Prevention of disease spread among returning travelers.	1987 (MenA) and 2000–01 (W135) outbreaks led to mandatory vaccination. Carriage rates dropped to 0.38% pre-Hajj and 1.10% post-Hajj, compared to 80% during previous outbreaks. No large-scale outbreaks since policy implementation.	[104]
National Immunization Days (NIDs)	2006—present	Egypt	Since Egypt was declared polio-free in 2006, two National Immunization Days (NIDs) are conducted annually as part of Egypt’s polio program. WHO, UNICEF, and USAID support the Egyptian Ministry of Health and Population (MoHP) to ensure that all children in Egypt, including vulnerable migrant populations, are protected against polio.	Maintain vaccination coverage over 97% Maintain polio-free status	Egypt achieved and maintained >97% OPV coverage annually through NIDs. Conducts 2 nationwide campaigns yearly targeting >15 million children under 5. No indigenous polio cases since 2004; certified polio-free in 2006.	[9]
Introduction of HPV Vaccine	2008—present	United Arab Emirates	In 2008, Abu Dhabi introduced the HPV vaccine into its immunization program, offering it free to girls aged 15–17 in public and private schools. By 2013, free immunization had been expanded to Emirati women aged 18–26. In 2019, the HPV vaccine was available to schoolgirls aged 13–14.	Significant reduction in cervical cancer prevalence.	HPV vaccine introduced in 2008 in Abu Dhabi; expanded to Dubai schools in 2023. Targeting girls aged 13–14, with full coverage for all Emirati and expatriate students in grade 8. Coverage rates exceeded 80% in Abu Dhabi during early implementation years.	[105]
Middle East Polio Outbreak Response Plan	2013–2015	Egypt, Iran, Iraq, Jordan, Lebanon, Palestine, Syria, and Turkey	Eight national governments partnered with the Global Polio Eradication Initiative (GPEI) to develop a multiphase response plan. Supplementary immunization activities (SIAs) were implemented, including health facility vaccinations, house-to-house visits, transit-point vaccinations, and mobile teams reaching vulnerable and hard-to-reach populations.	Vaccination of over 27 million children. Containment and interruption of the outbreak within 6 months of its identification.	38 WPV1 cases (36 in Syria, 2 in Iraq) >70 SIAs conducted across 8 countries Post-campaign OPV coverage rose to ≥93% in Syria Syria’s non-polio AFP rate rose from 1.7 (2013) to 4.0 (2014) OPV zero-dose children fell from 9% to 2% in Syria (2013–2015) Response interrupted transmission by April 2014	[106]
MERS-COV Vaccine Development	2015—present	SA and the UK	SA’s KAIMRC collaborated with the UK’s Oxford University in 2015 to develop the ChAdOX1 vaccine against MERS-COV, which was successful in mice and camels and can potentially protect humans and camels. Human phase 1 trials have begun in both countries.	Successful vaccine development and testing in animals. Initiation of human trials.	The vaccine showed complete protection in camels in preclinical studies. Phase I human trials began in 2018, with successfully demonstrated safety and immune response. As of 2022, KAIMRC initiated GMP production to prepare for larger-scale studies.	[107]
Routine Childhood Immunizations	1979—present	Jordan	Supported by UNICEF Jordan, the national immunization program, led by the Ministry of Health, routinely provides vaccinations for children residing in the country.	Jordan became polio-free in 1992. Decline in the rate of deaths and vaccine-preventable diseases (VPDs).	Successfully achieved the highest vaccination rates in the region, covering around 95% of the population.	[108]
Inclusive COVID-19 Vaccination Program	2021—present	Lebanon	In 2021, Lebanon’s COVID-19 vaccination campaign adopted an inclusive approach, covering all residents, including refugees, based on priority categories, such as morbidity and age, regardless of nationality or residency status. The initiative involved the Government of Lebanon, the private sector, the World Bank, UN agencies, and NGOs, with the World Bank reallocating USD 34 million from the Lebanon Health Resilience project and mobilizing additional funds for refugee vaccination.	Ensured equitable vaccine access for refugees. NGOs helped address vaccine hesitancy among refugees. Ensured transparency and fairness through an online pre-registration platform.	23% of non-Lebanese respondents indicated willingness to take the COVID-19 vaccine. 38,957 Palestinian and 45,195 Syrian refugees had pre-registered on the national vaccination platform.	[109]
Hayat Biotech Company	2021—present	UAE and China	Hayat Biotech is a joint venture between Sinopharm CNBG and G42, a company based in Abu Dhabi. The company focuses on life sciences, biotechnology, and vaccine production, supporting global health security and sustainability. They produced the Hayat-Vax vaccine, the first COVID-19 vaccine in the Middle East, and maintained a strategic stockpile to ensure readiness for future needs.	Produced the first COVID-19 vaccine in the Middle East, Hayat-Vax. Distributed 36 million doses across 64 countries. Ensured vaccine security with a production capacity of 200 million vials.	Production capacity of 200 million doses/year at Abu Dhabi KIZAD plant. 36 million doses exported to 64 countries as of 2022. UAE provided aid to 135 countries during the pandemic.	[110,111]
Sadeer City Vaccine and Vital Medicines Factory	2023—present	SA	The Saudi Authority for Industrial Cities and Technology Zones (Modon) has partnered with the Vaccine Industrial Company to establish a factory in Sadeer City for the manufacture of vaccines and vital medicines, with an investment of USD 133 million. This initiative aims to strengthen pharmaceutical security and localize vaccine production, including those for seasonal flu, COVID-19, chickenpox, pneumococcal, and meningitis.	Localize vaccine production. Potentially achieve 20% export of crucial vaccines. Create approximately 150 new jobs.	Investment of USD 133 million for production of COVID-19, flu, meningitis, and pneumococcal vaccines. Expected to create ~150 new jobs. Target to export 20% of critical vaccine output to regional markets.	[112]

The table outlines key vaccine initiatives in the Middle East, detailing their timelines, involved countries, summaries, key outcomes, and references; these initiatives demonstrate significant regional efforts to enhance immunization coverage, combat disease outbreaks, and ensure vaccine security.

**Table 2:** Socioeconomic Indicators and Immunization Funding Profiles.

Country	Income-Level	Global Peace Index (GDI) Ranking	Major Funding source	Gavi Eligibility	Reference
**Bahrain**	High Income	Medium 108	Fully government-funded	Not Eligible	[69,120,121,122]
**Kuwait**	High Income	High 35	Fully Government-Funded	Not Eligible	[69,120,121,123]
**Oman**	High Income	High 48	Government-Funded	Not Eligible	[69,120,121,124]
**Qatar**	High Income	High 21	Fully Government–Funded	Not Eligible	[69,120,121,125]
**Saudi Arabia**	High Income	Medium 119	fully government-funded	Not Eligible	[69,120,121,126]
**United Arab Emirate**	High income	Medium 75	fully government-funded	Not Eligible	[69,120,121,127]
**Yemen**	Low income	Very Low 162	Gavi, The Vaccine Alliance	Eligible	[69,120,121,128]
**Egypt**	Low-Middle Income	Medium 121	Government-Funded, excluding Campaigns which are funded by WHO, UNICEF and USAID	Not Eligible	[9,69,120,121]
**Iran**	High-Middle Income	Low 147	Government-Funded	Not Eligible	[69,120,121,129]
**Iraq**	High-Middle income	Very Low 154	Ministry of Health supported by the WHO and UNICEF	Not Eligible	[69,120,121,130]
**Jordan**	Low-Middle Income	High 62	Government-Funded	Not Eligible	[69,120,121,131]
**Syria**	Low Income	Very Low 161	Gavi, The Vaccine Alliance	Eligible	[69,120,121,132]
**Lebanon**	Low-Middle Income	Low 135	Government-Funded, Campaign Support Kuwait Fund & humanitarian donors via MOPH + WHO/UNICEF	Not Eligible	[69,120,121,133,134]
**Palestine**	Low-Middle Income	Low 134	Government-Funded, Campaign Support WHO, UNICEF, Gavi.	Not Eligible	[69,120,121,135,136]
**Turkey**	High-Middle Income	Low 147	Government-Funded	Not Eligible	[69,120,121,137]

## Data Availability

The data used in this study are publicly available from the sources cited in the References section.

## Supplementary Materials

The following supporting information can be downloaded at: https://www.mdpi.com/article/10.3390/vaccines13080806/s1. Additional file presents vaccination coverage across multiple vaccines for Middle Eastern countries from 2019 to 2024.

## Abbreviations

CEPI	Coalition for Epidemic Preparedness Innovations
DTP3	Diphtheria-Tetanus-Pertussis (3 doses)
EPI	Expanded Programme on Immunization
GCC	Gulf Cooperation Council
GPEI	Global Polio Eradication Initiative
GVAP	Global Vaccine Action Plan
HEPB3	Hepatitis B (3 doses)
HPV	Human Papillomavirus
IA2030	Immunization Agenda 2030
IMD	Invasive Meningococcal Disease
LICs	Low-Income Countries
MCV1	Measles-Containing Vaccine (1st dose)
MCV2	Measles-Containing Vaccine (2nd dose)
MENA	Middle East and North Africa
MERS-CoV	Middle East Respiratory Syndrome Coronavirus
MICs	Middle-Income Countries
NIDs	National Immunization Days
PCV3	Pneumococcal Conjugate Vaccine (3 doses)
ROTA	Rotavirus Vaccine
VPD	Vaccine-Preventable Disease
WHA	World Health Assembly
WUENIC	WHO/UNICEF Estimates of National Immunization Coverage
YF	Yellow Fever
